# Whole-Genome Sequences of a Cluster of 14 Unidentified Related *Veillonella* sp. Strains from Human Clinical Samples and Type Strains of 3 *Veillonella* Validated Species

**DOI:** 10.1128/MRA.01743-18

**Published:** 2019-03-21

**Authors:** Fabien Aujoulat, Michel R. Popoff, Laure Diancourt, Alexis Criscuolo, Hélène Jean-Pierre, Estelle Jumas-Bilak, Hélène Marchandin

**Affiliations:** a HydroSciences Montpellier, CNRS, IRD, Université de Montpellier, Montpellier, France; b Centre National de Référence des bactéries anaérobies et du botulisme, Institut Pasteur, Paris, France; c Institut Pasteur–Bioinformatics and Biostatistics Hub–C3BI, USR 3756 IP CNR, Paris, France; d CHU Montpellier, Laboratoire de Bactériologie, Montpellier, France; e HydroSciences Montpellier, CNRS, IRD, Université de Montpellier, CHU Montpellier, Laboratoire d’Ecologie Microbienne Hospitalière, Montpellier, France; f HydroSciences Montpellier, CNRS, IRD, Université de Montpellier, CHU Nîmes, Nîmes, France; Queens College

## Abstract

We report 17 draft genomes for 14 unidentified *Veillonella* sp. strains closely related in 16S rRNA gene-based phylogeny and type strains of 3 *Veillonella* species with the aims of deciphering relationships between related species, evaluating the accuracy of current thresholds for species delineation, and robustly describing new species in the genus.

## ANNOUNCEMENT

*Veillonella* spp. are anaerobic Gram-negative cocci and important representatives of the microbiota of humans and animals. Currently, 14 species in the genus *Veillonella* are validly described. In the absence of discriminative phenotypic characteristics, their identification requires molecular-based methods. However, all species are not discriminated by 16S rRNA gene (*rrs*) analysis because several pairs of species are closely related (≥99% of identical *rrs* nucleotides), such as V. denticariosi and V. rodentium, V. ratti and V. criceti, V. ratti and V. seminalis, and V. dispar and V. parvula (1–4). In addition, intrachromosomal heterogeneity between *rrs* copies (up to 1.43%) and intraspecific *rrs* variability that may surpass interspecific variability have been demonstrated in this genus (3, 5). This impairs the 16S rRNA gene-based identification of closely related species and suggests that applying the proposed revised threshold for a new species description, which is less than 98.7% of 16S rRNA gene identity, may not be adapted to the genus *Veillonella* (6). Therefore, molecular-based identification methods based on housekeeping genes such as *dnaK*, *rpoB*, and *gltA* were successively developed and used for the description of novel species in addition to or without associated DNA-DNA hybridization (DDH), which is the reference method for novel species description (2, 4).

We present draft genome sequences of 14 human clinical isolates and type strains of 3 *Veillonella* species that were not available at the time of our study. Isolates were recovered from human clinical samples from 14 patients attending the University Hospital in Montpellier, France. The study was approved by the institutional review board of the Nîmes University Hospital under the approval number 19.01.07. All strains were cultured on Columbia sheep blood agar (bioMérieux) at 37°C in an anaerobic jar with the AnaeroGen system (Oxoid Unipath) for 2 to 5 days (1, 4).

DNA was extracted with the MasterPure DNA purification kit (Epicentre Biotechnologies). Libraries were constructed with the Nextera XT DNA library preparation kit (Illumina, San Diego, CA) and sequenced on a NextSeq 500 instrument with a 2 × 150-bp paired-end protocol (on average, 1,372,528 read pairs per sample and ∼212× sequencing depth; Table 1). All sequenced paired-end reads were clipped and trimmed with AlienTrimmer v. 0.4.0 (7), corrected with Musket v. 1.1 (8), and subjected to a digital normalization procedure with khmer v. 1.3 (9). For each sample, the remaining processed reads were assembled and scaffolded with SPAdes v. 3.11.0 (10). All programs were used with their default settings. Strains and characteristics of their whole-genome sequences (WGSs) are presented in Table 1.

**TABLE 1:** Characteristics of the 17 *Veillonella* sp. draft genome sequences^*a*^

Species	Strain	Accession no.	BioSample no.	No. of paired-end reads	Coverage (×)	Genome size (bp)	No. of contigs	*N*_50_ (bp)	G+C content (%)
*V. ratti*	ATCC 17746^T^	RQUX00000000	SAMN10465589	1,163,748	156	2,236,286	49	95,000	42.8
*V. caviae*	DSM 20738^T^	RQUY00000000	SAMN10465590	1,391,318	211	1,963,997	57	83,095	38.4
*V. seminalis*	ADV 4313.2^T^ = CIP 107810T	RQUZ00000000	SAMN10465591	1,399,442	184	2,252,690	27	152,260	42.2
*Veillonella* sp.	CHU110 = 14/09/02‐B‐6149	RQVA00000000	SAMN10465592	973,834	155	1,881,921	43	107,364	39.4
*Veillonella* sp.	Va140 = CHU594 = 03/11/05‐B‐4237	RQVB00000000	SAMN10465593	1,206,819	199	1,822,704	23	147,662	39.3
*Veillonella* sp.	Va138 = CHU732 = 06/09/06‐B‐3252	RQVC00000000	SAMN10465594	740,997	112	1,972,307	42	104,904	39.5
*Veillonella* sp.	Va143 = CHU740 = 09/10/06‐B‐1508	RQVD00000000	SAMN10465595	1,518,558	240	1,893,839	35	130,014	39.3
*Veillonella* sp.	3891 = 12/03/14‐B‐3204	RQVE00000000	SAMN10465596	1,295,195	205	1,890,765	38	161,698	39.4
*Veillonella* sp.	3913 = 09/03/14‐B‐7013	RQVF00000000	SAMN10465597	698,249	118	1,770,519	15	394,883	39.4
*Veillonella* sp.	3627 = CHU4040 = 99141203627	RQVG00000000	SAMN10465598	1,308,145	226	1,738,782	34	132,446	39.3
*Veillonella* sp.	CHU3735 = CNR 79/14 = 14/01/14‐B‐2378	RQVH00000000	SAMN10465599	1,123,247	190	1,776,932	51	93,431	39.4
*Veillonella* sp.	3960 = CHU4076 = 99141423960	RQVI00000000	SAMN10465600	746,487	120	1,860,088	69	60,297	39.7
*Veillonella* sp.	3310 = CHU5981 = 99162773310	RQVJ00000000	SAMN10465601	1,640,876	258	1,870,378	16	311,068	39.6
*Veillonella* sp.	Va137 = 19/07/06‐3404	RQVK00000000	SAMN10465602	1,966,481	310	1,902,403	36	128,606	39.6
*Veillonella* sp.	Va139 = 10/01/06‐3360	RQVL00000000	SAMN10465603	2,226,458	303	2,172,276	76	67,981	39.5
*Veillonella* sp.	Va141 = 20/06/06‐2457	RQVM00000000	SAMN10465604	2,034,756	333	1,806,031	12	229,161	39.2
*Veillonella* sp.	Va142 = 08/06/05‐3381	RQVN00000000	SAMN10465605	1,898,363	281	1,969,382	66	75,004	39.1

a BioProject number, PRJNA506647.

Because genomic sequencing is being progressively incorporated into the taxonomy of *Bacteria* (11) and considering that it was not used before in the genus *Veillonella*, taxogenomic metrics such as *in silico* DDH, average nucleotide identity, and phylogenomics will be valuable for (i) deciphering intrageneric relationships between related species and between strains affiliated with a species or of unknown taxonomic status, (ii) evaluating the accuracy of current thresholds for species delineation, (iii) supporting reappraisal of the taxonomy, (iv) robustly describing new species, and (v) proposing minimal standards for description of new species in the genus *Veillonella*. Besides taxonomic purposes, WGS will also allow the characterization of clinically relevant gene content, particularly virulence and antibiotic resistance genes, in *Veillonella* spp. considered opportunistic human pathogens.

### Data availability.

This whole-genome shotgun project was deposited at DDBJ/ENA/GenBank under the accession numbers RQUX00000000 to RQVN00000000 as listed in Table 1 (BioProject number PRJNA506647; BioSample numbers SAMN10465589 to SAMN10465605). The versions described in this paper are versions RQUX01000000 to RQVN01000000. The Sequence Read Archive accession numbers are SRX5189907 to SRX5189923.
